# Supplementary material for: Cellular entry and uncoating of naked and quasi-enveloped human hepatoviruses
Source: eLife. 2019 Feb 25;8:e43983. doi: 10.7554/eLife.43983 (PMC6422491; doi:10.7554/eLife.43983)
Supplement: Supplementary file 1. [file elife-43983-supp1.docx]

**eLife 43983 Rivera-Serrano et al. Supplementary File 1: Nucleotide sequences of sgRNAs, gene-specific RT-PCR primers and siRNAs.**

**sgRNA CRISPR/Cas9 All-in-One Lentivectors**

| sgRNA target | Target sequence | abm cat. no. |
| --- | --- | --- |
| Scrambled | GCACTCACATCGCTACATCA | K010 |
| *ITGB1* | ATACAAGCAGGGCCAAATTG | K1104806 |
| *ITGA1* | AATGACTTTCAGCGGCCCGG | K1103006 |
| *ITGA4* | TGGGAAGCGAGGCGCGAACC | K1103406 |
| *ITGAV* | CCGCGCCTTCAACCTAGACG | K0007206 |
| *PLA2G16* | CTGCAGCAAAATCATCCAGC | K1660508 |

**Gene-specific RT-PCR primers**

| Gene | Forward primer, 5’ → 3’ | Reverse primer, 5’ → 3’ |
| --- | --- | --- |
| *ALIX (PDCD6IP)* | GGAAGAGCAGGCCCAGTA | CGAAAGCATCCTTCCAGGT |
| *CHMP1B* | GACGATGGGCAAGGTGAC | TCATCGTGTCTTCCATTTGC |
| *CHMP2A* | AGAAGATGGCCAAGCAAGG | GCCATCGAGTTGTTGGACTT |
| *PLA2G16* | ACAAGGCCATCGTGAAGAAG | CTCGCAGTTCTCACTGGTCA |
| *LGALS8* | AAATCGCACCCAGAACTGTC | AGCTTTTGGCATTTGCATTC |

**SMARTpool ON-TARGET siRNA sequences**

| mRNA target (Dharmacon no.) | siRNA #1, 5’ → 3’ | siRNA #2, 5’ → 3’ | siRNA #3, 5’ → 3’ | siRNA #4, 5’ → 3’ |
| --- | --- | --- | --- | --- |
| Non-target (D-001810) | UGGUUUACAUGUCGACUAA | UGGUUUACAUGUUGUGUGA | UGGUUUACAUGUUUUCUGA | UGGUUUACAUGUUUUCCUA |
| *CLTC* (L-004001) | GAAAGAAUCUGUAGAGAAA | GCAAUGAGCUGUUUGAAGA | UGACAAAGGUGGAUAAAUU | GGAAAUGGAUCUCUUUGAA |
| *AP2M1* (L-008170) | UAUAUGAGCUGCUGGAUGA | GAAGAGCAGUCACAGAUCA | CGUGAUGGCUGCCUACUUU | GGAGGCUUAUUCAUCUAUA |
| *DNM2* (L-004007) | GGCCCUACGUAGCAAACUA | GAGAUCAGGUGGACACUCU | CCGAAUCAAUCGCAUCUUC | GAGCGAAUCGUCACCACUU |
| *CAV1* (L-003467) | CUAAACACCUCAACGAUGA | GCAAAUACG­UAGACUCGGA | GCAGUUGUACCAUGCAUUA | GCAUCAA­CUUGCAGAAAGA |
| *FLOT1* (L-010636) | GCAGAGAAGUCCCAACUAA | GUGGUUAGCUACACUCUGA | GAUCAGUGGUCCCUUGACU | GAAGACGGAGGCUGAGAUU |
| *ARF6* (L-004008) | CGGCAUUACUACACUGGGA | UCACAUGGUUAACCUCUAA | GAGCUGCACCGCAUUAUCA | GAUGAGGGACGCCAUAAUC |
| *VCAM1* (L-013351) | GGAGAUAGACUUACUGAAA | CAAAGUUGGCUCACAAUUA | GAAGGAUGCGGGAGUAUAU | CCUUGGAGCCUCAAAUAUA |
| *ICAM1* (L-003502) | GGUAGCAGCCGCAGUCAUA | GAGCCAAGGUGACGCUGAA | CGGCUGACGUGUGCAGUAA | GAAGAUAGCCAACCAAUGU |
| *ITGB1* (L-004506) | GUGCAGAGCCUUCAAUAAA | GGUAGAAAGUCGGGACAAA | UGAUAGAUCCAAUGGCUUA | GGGCAAACGUGUGAGAUGU |
| *ITGB3* (L-004124) | GCAGUGAAUUGUACCUAUA | GAAGAACGCGCCAGAGCAA | GCCAACAACCCACUGUAUA | CCAGAUGCCUGCACCUUUA |
| *ITGA1* (L-008516) | AGACAAAUAUCACGAAGUU | GGAUUUAAAUGGUGACGGU | CAGCUAUAACCGAGGAAAU | CCUUCUACAUGUUGGACAA |
| *ITGA2* (L-004566) | GAACGGGACUUUCGCAUCA | GAAACGCCCUUGAUACUAA | GUUCAGACCUACUAAGCAA | AAACAAGGCUGAUAAUUUG |
| *ITGA3* (L-004571) | CCAAGGAAACCUCUAUAUU | GCGCAAGGAGUGGGACUUUA | GGAGUGGCCCUACGAAGUC | GUGUACAUCUAUCACAGUA |
| *ITGA4* (L-005189) | GACAAGACCUGUAGUAAUU | GCAUAUAUAUUCAGCAUUG | CAGAGUGACUGUAGCAAUA | AGAGACAGUUGGAGUUAUA |
| *ITGA5* (L-008003) | GAACGAGUCAGAAUUUCGA | UCACAUCGCUCUCAACUUC | ACACGUUGCUGACUCCAUU | CAAACGCUCCCUCCCCAUAU |
| *ITGA6* (L-007214) | GGAUCGAGUUUGAUAACGA | GGAUAUGCCUCCAGGUUAA | GAAAGGGAUUGUUCGUGUA | ACAGAUAGAUGAUAACAGA |
| *ITGA7* (L-008004) | GAGGAGUACUCAGCUGUGA | GGCCCUCAAUAGCUACUUA | UAUGAUUGGUCGCUGCUUU | CAAGGCCAGUCGCUCAGAA |
| *ITGA8* (L-010699) | GAAAGUCAGGUCACGAUUA | ACAGAAAGAUCAGAGUUAA | GCAUAGCAAUUAAGACAUC | GGACGCUCUUCCUUGAUAA |
| *ITGA9* (L-008005) | GUGCAGAGAUGUUUCAUGU | AAAUACAGCCCUUCAGUGA | GAAGAAAGUCGUACUAUAG | GAUAGUCCCUCAGUACUCA |
| *ITGAV* (L-004565) | CCUCUGACAUUGAUUGUUA | CCGAAACAAUGAAGCCUUA | GAACAUGUCCUCCUUAUAC | GUUCACGCCUGCUAACAUU |
| *TSPAN8* (L-010219) | GCAAUAUGGGUACGAGUAA | GGACAUAUUGAUUGCUGUA | GAUACUGGGUUUGGUGUUU | GGGAUGCUGCGGUGGUAUA |
| *Rab5A* (L-004009) | AGAGUCCGCUGUUGGCAA | GGAAGAGGAGUAGACCUUA | UGACACUACAGUAAAGUUU | UGACACUACAGUAAAGUUU |
| *Rab7A* (L-010388) | CUAGAUAGCUGGAGAGAUG | AAACGGAGGUGGAGCUGUA | GAUGGUGGAUGACAGGCUA | GGGAAGACAUCACUCAUGA |
| *Rab11A* (L-004726) | GCAACAAUGUGGUUCCUAU | CAAGAGCGAUAUCGAGCUA | GUGCAGUGCUGUCAGAACA | GAGAUUUACCGCAUUGUUU |
| *NPC1* (L-008047) | GGACAACUAUACCCGAAUA | GAAGAAGCCCGACUUAUA | GCGAACGGCUUCUAAAUUU | GAUGAGACCAAUUGUGAUA |
| *LIPA*/*LAL* (L-004043) | CGUUUGCACUCAUGUCAUA | UGUCUAGAGUGGAUGUAUA | CAAAUUAGGACGAUUACCA | GAACCAUUCUGACAAAGGU |
| *LAMP1* (L-013481) | CAACAGAGUAACUAUCGAA | GGCUUAGGGUCCUGUCGAA | AAGUACAACGUGAGCGGCA | AUGAACAACGUGACCGUAA |
| *ALIX* (L-004233) | CAGAUCUGCUUGACAUUUA | UCGAGACGCUCCUGAGAUA | GCGUAUGGCCAGUAUAAUA | GUACCUCAGUCUAUAUUGA |
| *CHMP1B* (L-004698) | CAAAUUCGAGCACCAGUUU | GUCGAUGGCUGGUGUGGUU | CCUUCGGGAUCAAGUGUGA | CAUGGAAGUUGCGAGGAUA |
| *CHMP2A* (L-020247) | CAAAAGACUUGGUGCGCAC | UGAUGGAGUUUGAGCGGCA | CAGAUGAGCUGUCGAACCU | GCGCAAGUUUGUAUUGAUG |
| *PLA2G16* (L-019760) | GCGAUGGAUAUGUGGUUCA | UCAAGAAACAAAGCGACAAA | ACAAGUACCAGGUCAACAA | UGAAUGAGCUGCGCUAUGG |
| *LGALS8* (L-010607) | GAACUGUCGUCGUUAAAGG | GAGCCGAUGUGGCCUUUCA | GUAAGGAGCUGGUAGCCUA | UAUCAUCUAUAACCCGGUA |
